# The driving role of extracellular polymeric substances in the bioelectrical conversion of petroleum hydrocarbons by rhizosphere microbial fuel cells: bioelectricity production, substrate bioconversion, microbial function and their network correlation

**DOI:** 10.1186/s40643-025-00922-4

**Published:** 2025-07-25

**Authors:** Lanmei Zhao, Yuru Wen, Lyu Can, Long Meng, Jian Liu, Dong Zhao

**Affiliations:** 1 College of Chemical and Biological Engineering, Shandong University of Science and Technology, Qingdao, 266590 China; 2 Tobacco Research Institute of Chinese Academy of Agricultural Sciences, Qingdao, 266001 China; 3 Beijing Life Science Academy, Beijing, 102209 China; 4 Sinopec Shengli Petroleum Administration, Dongying, 257000 China

**Keywords:** Bioelectricity generation, Bioelectrical conversion, Microbial function, Network correlation, Extracellular polymeric substances

## Abstract

Extracellular polymeric substances (EPS), are crucial components of biofilms that drive the bioelectrical conversion of petroleum hydrocarbons (PHCs), but their role has not been adequately addressed. This research explores the driving role of EPS in bioelectrical PHC conversion by rhizosphere microbial fuel cells (MFCs). We found that current density, output voltage, coulombic efficiency, power density, current stabilization time, metabolite volatile fatty acid (VFA) production and PHC biodegradation ratio initially increased and then decreased with rising initial EPS level (0-128 mg·g^− 1^), peaking at 64 ± 1 mA·m^− 2^, 8.04 ± 0.16 V, 60.9 ± 1.2%, 129 ± 3 mW·m^− 2^, 23 ± 1 days, 1.77 ± 0.04 g·kg^− 1^ and 66.7 ± 1.5%, respectively. Fluorescence intensity of proteins having tyrosine-tryptophan demonstrated a continuous enhancement, consistent with increased biofilm thickness. Within an appropriate range of initial EPS levels (0–64 mg·g⁻¹), bioelectricity generation and PHC bioconversion enhanced as the EPS content rose in mature biofilms. However, excessive EPS addition could increase biofilm thickness to 0.48 mm, which in turn reduced biofilm activity and overall system performance. The abundances of electrochemically active and PHC-degrading bacteria presented an initial increase followed by a subsequent decrease as the initial EPS level rose, highlighting that EPS at the optimal level enriched and activated these functional bacteria. The positive correlations between the relative abundances of these bacteria and various metrics of bioelectricity generation and PHC bioconversion underscored the critical role of EPS in shaping microbial community structure and enhancing electron transfer efficiency through biofilm formation and stabilization. These findings not only provide a critical theoretical foundation and novel ideas to promote the conversion of PHC into renewable bioenergy but also highlight the potential scalability and environmental benefits of this technology in the field of clean remediation of PHC-polluted soils and recovery of bioenergy. Integrating EPS-driven MFCs with other renewable energy technologies will offer promising opportunities to develop hybrid systems that generate clean energy while mitigating environmental pollution. Furthermore, this approach also has the potential as biosensors for the real-time detection of PHCs, thus contributing to broadening its application in environmental monitoring.

**Graphical Abstract:**

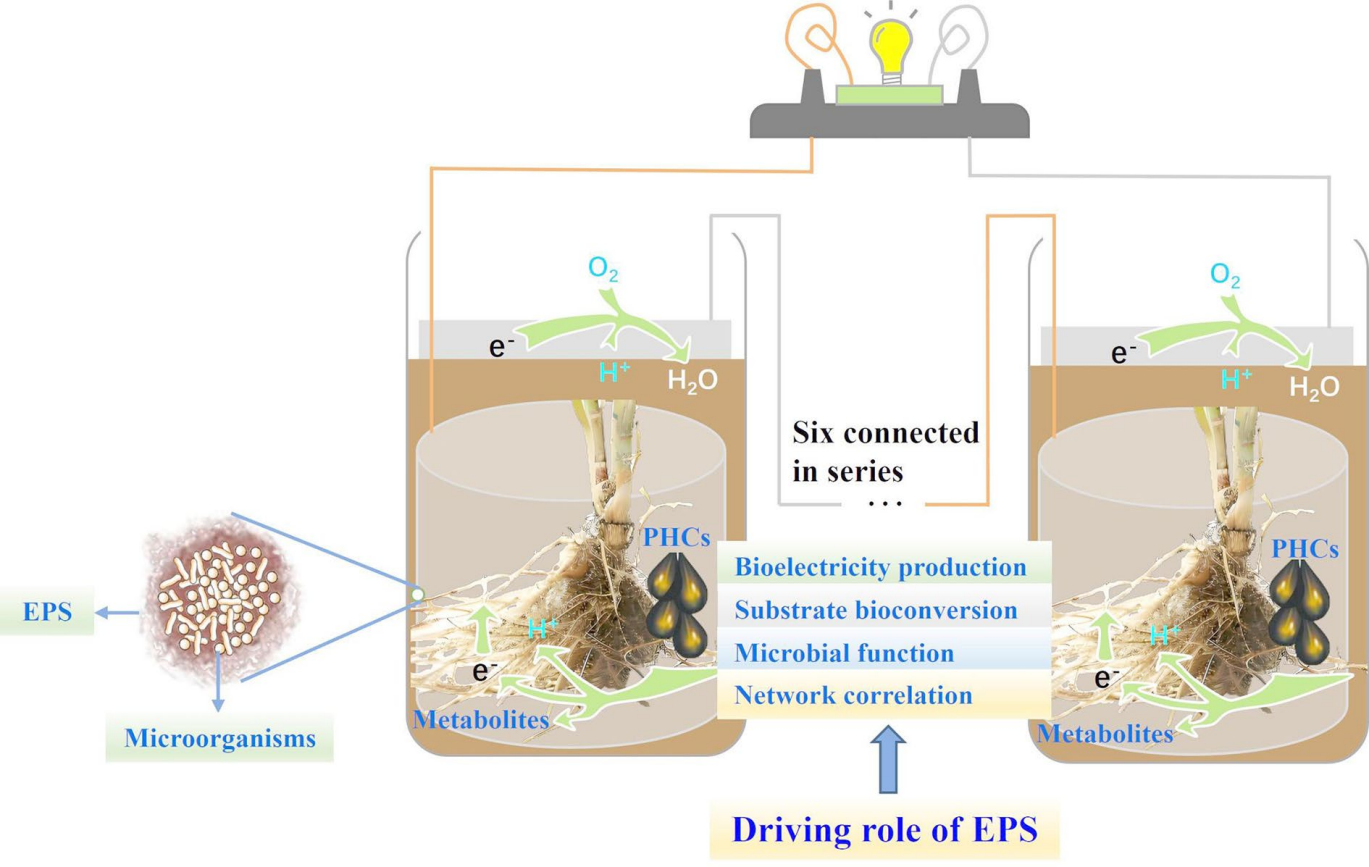

## Introduction

With the continuous exploitation of petroleum resources in recent decades, the wetlands of the Yellow River Delta—recognized as the most extensive, intact, and youngest wetlands—have suffered significant petroleum pollution. This environmental challenge underscores the urgent need for innovative technologies that can simultaneously achieve wetland purification and energy recovery. Among such technologies, sedimentary microbial fuel cells (SMFCs) have emerged as an attractive solution for in-situ remediation of petroleum-contaminated sediments and clean bioenergy recovery. Although still in their infancy, SMFCs offer several advantages, including energy saving, accelerated decontamination, self-sustained operation, easy regulation and environmental friendliness (Zhao et al. 2025). SMFC systems typically consist of an anode buried in the sediment and a cathode exposed to an aerobic surface, enabling in-situ remediation through microbial electrochemical processes. The efficiency of these processes is regulated by the interactions between electrodes and microorganisms, particularly the biofilm formation on the anode surface (Huang and Puma 2022). Anodic biofilms play a central role in SMFC performance, with their three-dimensional structure, strong cell viability and conductive components driving bioelectricity generation (Wu et al. 2022). Within these biofilms, EPS are critical, not only providing structural support but also acting as electron mediators that enhance extracellular electron transfer (EET) efficiency (Guo et al. 2022).

EPS play a crucial role in the function of microbial fuel cells (MFCs), with recent studies highlighting their diverse contributions to bioelectrochemical systems. Acting as vital carbon and energy sources for electroactive bacteria, EPS influence voltage output and biofilm conductivity through redox-active proteins (He et al. 2025; Ontita et al. 2024). On the other hand, bioelectrochemical stimulation has been shown to improve conductivity by promoting the secretion of redox-active proteins (Li et al. 2024), indicating that EPS composition directly impacts system performance. The relationship between EPS composition and electron transfer efficiency is particularly complex, as redox-active proteins accelerate electron transfer (Quan et al. 2024; Wang et al. 2024), while polysaccharides exhibit context-dependent effects: excessive accumulation can hinder electron transport by increasing biofilm thickness, yet under certain conditions, such as higher anode potentials, they may support metabolic activity and biofilm maturation (Xu et al. 2024). Furthermore, elevated protein content in EPS has been linked to enhanced electron exchange capacity, whereas polysaccharides can act as either structural scaffolds or barriers depending on their spatial distribution (Qi et al. 2024; Yang et al. 2025). Recent studies have explored the role of EPS in MFCs, highlighting their contributions to electron transfer and system performance through redox-active proteins and polysaccharides. However, these studies often focus on isolated aspects of EPS composition and function without addressing how varying initial EPS levels influence the dynamic evolution of MFC systems. The interplay between EPS components, biofilm development, and environmental factors remains poorly understood, particularly under conditions of stress or fluctuating anode potentials. The dynamic interactions between EPS levels and biofilm development, as well as between EPS levels and MFC performance are still poorly understood, particularly under extreme environmental conditions.

Previous studies have preliminarily explored the link between EPS and bioelectrical PHC conversion. For instance, the production of EPS within anodic biofilms was found to increase with crude oil levels, correlating with higher metabolite production, power density and PHC degradation ratio (Zhao et al. 2023). Additionally, the presence of EPS was shown to significantly enhance the biodegradation of PHCs, with the degradation ratio doubling compared to that without EPS, and improving the breakdown of non-volatile hydrocarbons (C16-C24) (Meng et al. 2024). Furthermore, the bioconversion of PHC under aerobic conditions resulted in elevated levels of EPS components, such as humic acids, proteins and polysaccharides, compared to anaerobic bioreactors where SO_4_^2−^ and NO_3_^−^ served as electron acceptors (Zhao et al. 2019). Proteins having tyrosine-tryptophan, which are one of EPS components, have been shown to mutually reinforce biofilm formation and PHC metabolism, facilitating bioelectricity generation (Zhao et al. 2025; Zhao and Zhao 2022).

Despite these advances, a critical research gap exists regarding the role of EPS in the dynamic interactions among biofilm development, PHC bioconversion and bioelectricity generation in MFCs, particularly under varying environmental conditions and stresses such as carbon limitation, high salinity and complex pollutants—conditions characteristic of the extremophilic microorganisms in the Yellow River Delta wetlands. While studies have identified the contributions of EPS to electron transfer efficiency and PHC degradation, the impact of initial EPS levels on biofilm evolution and system performance remains unclear. Furthermore, previous researches have primarily focused on the general properties of EPS or their role in microbial adhesion and electron transfer, neglecting the diversity of EPS secretion and its driving role in anodic biofilm formation and PHC bioconversion in extremophilic environments. This lack of understanding may stem from the complexity of EPS interactions with microbial communities and their adaptation to fluctuating anode potentials or stress conditions. To address this, we propose the hypothesis: “Variations in initial EPS levels influence the dynamic evolution of MFC systems by regulating biofilm development and EET, thereby influencing PHC degradation efficiency and bioelectricity generation.”

Our research directly addresses these limitations by investigating the driving role of different initial EPS levels in the evolution dynamics of rhizosphere MFC systems for bioelectrical PHC conversion, focusing on bioelectricity production, substrate bioconversion, microbial function and their network correlation. This approach highlights the novelty and necessity of our work, with the potential to bring more efficient bioelectrochemical systems for environmental remediation and bioenergy recovery.

## Materials and methods

### Experimental materials

Vegetation colonization is a crucial factor in the changes of ecological functions and microbial community structure in the wetlands of Yellow River Delta. Reed (*Phragmites australis*), as one of the typical vegetation, is distributed in low-salinity upland far from seawater (Sekaran et al. 2021). The rhizosphere microbial communities formed by reeds in PHC-polluted wetlands have typical characteristics of substrate preference, anaerobicity and salinity tolerance, so reed and its rhizosphere soil were selected to construct the rhizosphere MFC system in this study. The samples of uncontaminated test plant and wetland soil were collected from the transition zone of reed vegetation in the wetlands of Yellow River Delta. The PHCs used in this study, sourced from Shengli Oilfield in Dongying, China, classified as light oil, was primarily composed of 89.2% aliphatic hydrocarbons (with C6-C18 alkanes as the dominant fraction), 10.4% aromatic hydrocarbons, and trace amounts of other organic compounds (Zhao et al. 2025). The PHC content of some reed rhizosphere reached up to approximately 5 g/kg in the wetlands of Yellow River Delta (Zhao et al. 2025), so their content was set at 5 g/kg in this study. To create PHC-polluted wetlands, 20 g of PHCs was mixed with 600 mL of dichloromethane and combined with 4 kg of wetland soil. The mixture was then stirred thoroughly every 12 h and left to air dry for two days to allow the dichloromethane to fully evaporate.

### Experimental equipments and procedures

A cylindrical plexiglas with a diameter of 30 cm and a height of 40 cm was used to construct a reaction unit for the rhizosphere MFC systems. A reed and 4 kg of PHC-polluted wetland soil (5 g/kg) were planted in a reaction unit. The carbon fiber brushes were used as the anode, and a cylindrical anode with an area of 0.1256 m^2^ was placed vertically on the outer periphery of the rhizosphere soil of reed plant under test, and the carbon felts as the cathode (0.02 m^2^) were placed horizontally on the surface of the wet soil in contact with the air. The electrodes were pretreated using ultrasonic cleaning before use to remove stubborn contaminants and fine particles. The heating tape was wrapped around the reaction unit and the temperature was set to 30 °C. Thus, a single reaction unit was constructed. Six identical reaction units were connected in series and externally connected to a 1000 Ω resistor to form a rhizosphere MFC system (R0). Biofilm maturity was considered achieved when both biofilm thickness (observed through SEM) and bioelectricity production (power density and current density) stabilised over time, with less than a 10% variation observed in these parameters. After the anode biofilms matured, they were removed using a sampler to extract the EPS secreted by the microorganisms in the anode biofilms. The measured EPS level in six reaction units was 128 ± 14 mg/g dry soil. The EPS were diluted step by step in a gradient of 2^0^, 2^1^, 2^2^, 2^3^, 2^4^, 2^5^ and 2^6^ using 0.9% NaCl solution, and the obtained EPS level was recached 128, 64, 32, 16, 8, 4 and 2 mg/g (based on the average values), respectively. Equal amounts of clean water and diluted EPS, in increasing order of initial EPS level from low to high, were added to eight groups of rhizosphere MFC systems (R1, R2, R3, R4, R5, R6, R7, and R8) in the same way as R0 before the startup, and each group was set up in three parallels and restarted. The initial EPS levels added to R1, R2, R3, R4, R5, R6, R7 and R8 were 0, 2, 4, 8, 16, 32, 64 and 128 mg/g, respectively. All MFC systems operated for 100 days. When the anode biofilm in the eight groups of rhizosphere MFC systems matured, PHC biodegradation and bioelectricity production performance were evaluated. The anodic mature biofilm samples were then collected for subsequent testing and analysis.

Eight groups of rhizosphere MFC systems (R1 to R8) were established, representing a stepwise gradient of initial EPS levels (0, 2, 4, 8, 16, 32, 64 and 128 mg/g). This gradient was necessary to investigate the effect of varying EPS levels on PHC bioconversion and bioelectricity generation performance. The inclusion of eight groups ensured a comprehensive analysis across a wide range of EPS levels, which was critical for understanding the relationship between initial EPS level and MFC system performance.

### Test and analytical methods

#### Performance test of rhizosphere MFCs

Regarding the determination of metabolite VFA productions in R1-R8, high performance liquid chromatography was employed (Zhao et al. 2023). The main operational conditions were as follows. 10 mL of sludge-water mixture were subjected to centrifugation at 3000 g for 10 min, after which the obtained solution was filtered through a 0.22 μm filter membrane. 10 µL of the filtered water sample was used for performing the test. The mobile phase comprised a solution of 9 mmol·L^− 1^ H_2_SO_4_, with a flow rate of 0.6 mL·min^− 1^. The chromatographic column was employed as an Alltech OA-1000 organic acid column at 55 °C, and the wavelength of the detector was set to 210 nm. UV-visible spectrophotometry at 225 nm was used to determine the concentration of PHCs in the organic phase, following the extraction of PHCs from the sludge-water mixture with petroleum ether on three occasions (Zhao et al. 2023). The polarization curves and power density curves of R1-R8 were obtained by disconnecting the external circuit for a period of two hours and subsequently employing a potentiostat at 10-minute intervals. The output voltage (U) was measured by a multichannel voltage recorder, and the current (I) was continuously monitored using a precision multimeter and a data acquisition system (Zhao and Zhao 2022). The resulting values for the power density and Coulombic efficiency were calculated using the formulas P = UI/A and CE = It/(Ne∙F), respectively. In both formulas, A represented the surface area of anode, and Ne was the total number of electrons released from PHC biodegradation, and F was the Faraday constant.

#### EPS extraction and detection in anodic mature biofilms

Different EPS separation and extraction methods could affect the EPS properties and cause the differences in protein fractions, which played an important role in the electroactivity of biofilms. Heating extraction method is more damaging to microbial cells, and EDTA and ultrasonic extraction methods are less damaging to microbial cells. Ultrasonic extraction method could help to extract redox-active proteins in EPS, and the microbial cells were viable after ultrasonic extraction. Therefore, ultrasonic extraction method was adopted to extract EPS from anodic mature biofilms in this study (Yu et al. 2008). 3 g (wet weight) of biofilm samples from R1-R8 were suspended in 50 mL of 0.9% NaCl solution, and ultrasonicated at 20 KHZ for 2 min, then shaken horizontally at 150 g for 10 min, and ultrasonicated again for 2 min, and finally centrifuged at 5000 g for 15 min. The supernatant was collected and filtered using a filter membrane with a pore size of 0.22 μm, to obtain the loosely bound EPS (LB-EPS). The filter cake was resuspended to 50 mL with 0.9% NaCl solution, and ultrasonicated at 20 KHZ for 10 min, and centrifuged at 5000 g for 20 min to collect the supernatant. The filter cake obtained for the second time was resuspended and precipitated with saline, and centrifuged at 5000 g for 15 min, and the supernatant was collected. The filter cake obtained after repeating the above operation for the third time. The supernatants obtained above were merged and filtered using a filter membrane with a pore size of 0.22 μm, to obtain tightly bound EPS (TB-EPS). The TB-EPS type and density were detected by the fluorescence analysis technique (three-dimensional excitation-emission matrix, 3D-EEM).

#### Functional microbial testing and analysis of anode mature biofilms

The anode mature biofilm samples in R1-R8 were sent to Major Bio-group in Shanghai for high throughput sequencing. The main sequencing and analysis steps were shown below. After genomic DNA extraction was completed, the extracted DNA was detected by 1% agarose gel electrophoresis. Specific barcoded primers were synthesized according to the designated sequencing region. PCR was performed using TransGen AP221-02 TransStart^®^ FastPfu DNA Polymerase for amplification. PCR products were recovered by cutting the gel using AxyPrepDNA Gel Recovery Kit (AXYGEN) and eluted with Tris-HCl buffer; detected by 2% agarose gel electrophoresis. Library construction and sequencing were performed on the Illumina PE300 platform. Based on the obtained raw sequencing data, a series of analytical results such as species annotation and evaluation, species composition, species difference, sample comparison, etc. were obtained through quality control splicing, OUT clustering, data analysis and information extraction.

#### *The network correlation method of functional microorganisms with bioelectricity generation*, PHC *bioconversion and EPS intensity*

The Spearman’s rank correlation coefficients of functional microorganisms with PHC biodegradation ratio, metabolite VFA production, current density, output voltage, coulombic efficiency, maximum power density, current stability time, biofilm thickness and fluorescence regional integration (FRI) of EPS were calculated using R 4.4.0, and *P* < 0.05 and Spearman’s|RHO|>0.6 was selected for the correlation network analysis by Gephi 0.9.2.

#### Statistical analysis of the data

Statistical analysis was performed using SPSS 29 software to determine data significance. Tukey’s Honest Significant Difference test and Analysis of Variance were employed to identify significant differences at a confidence level of *p* < 0.05. The results were indicated using lowercase letters in the figures of this study.

## Results and discussion

### Bioelectricity generation and PHC bioconversion in reed rhizosphere MFCs with different initial EPS levels

**Fig. 1:**
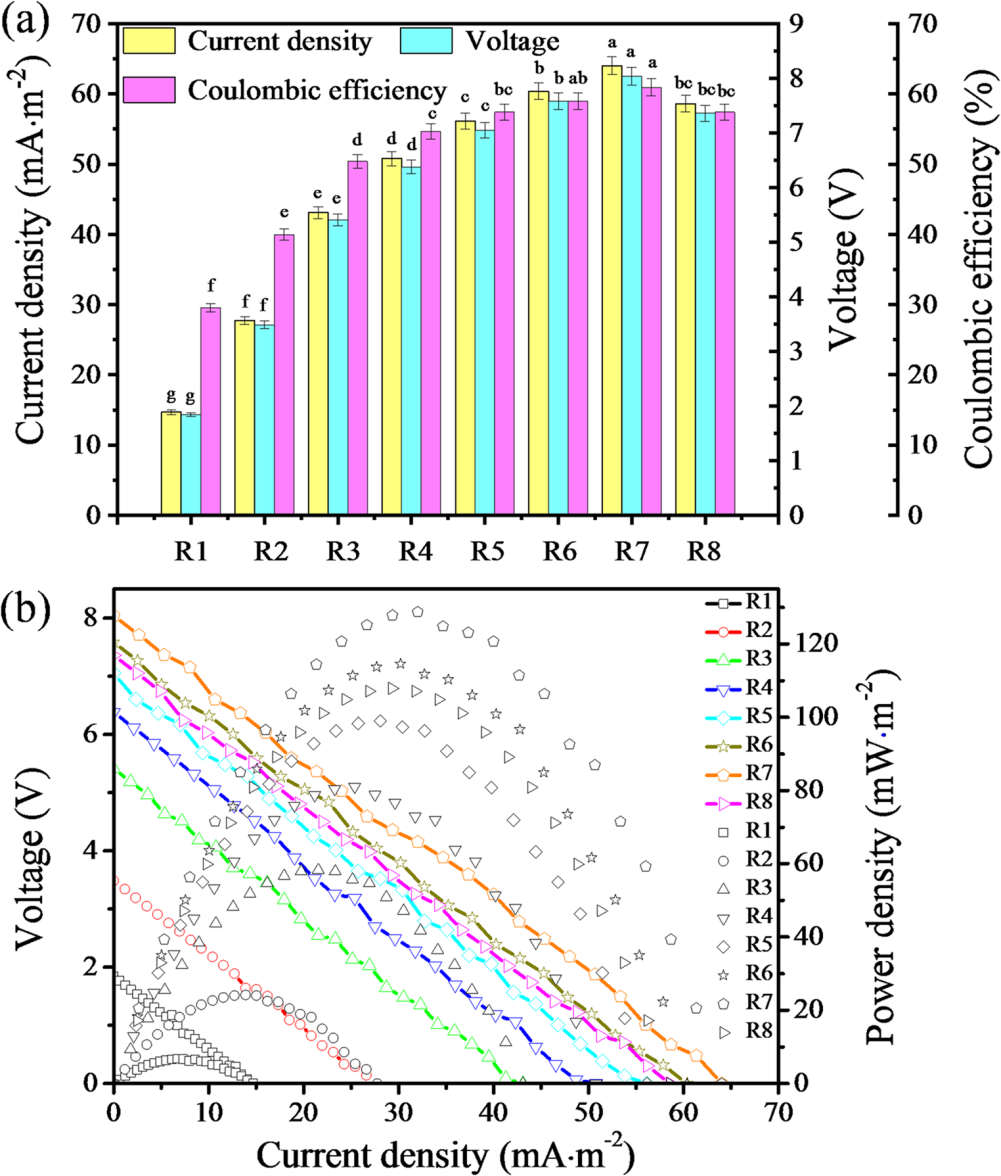
(**a**) Current density, output voltage, coulombic efficiency, and (**b**) polarization curve of reed rhizosphere MFCs with different initial EPS levels. R1-R8 represented the reed rhizosphere MFCs with the initial EPS level from 0 to 128 mg·g^− 1^. Values in the same indicator with different letters indicated significant differences (*P* < 0.05) by Tukey’s honestly significant difference test

**Fig. 2:**
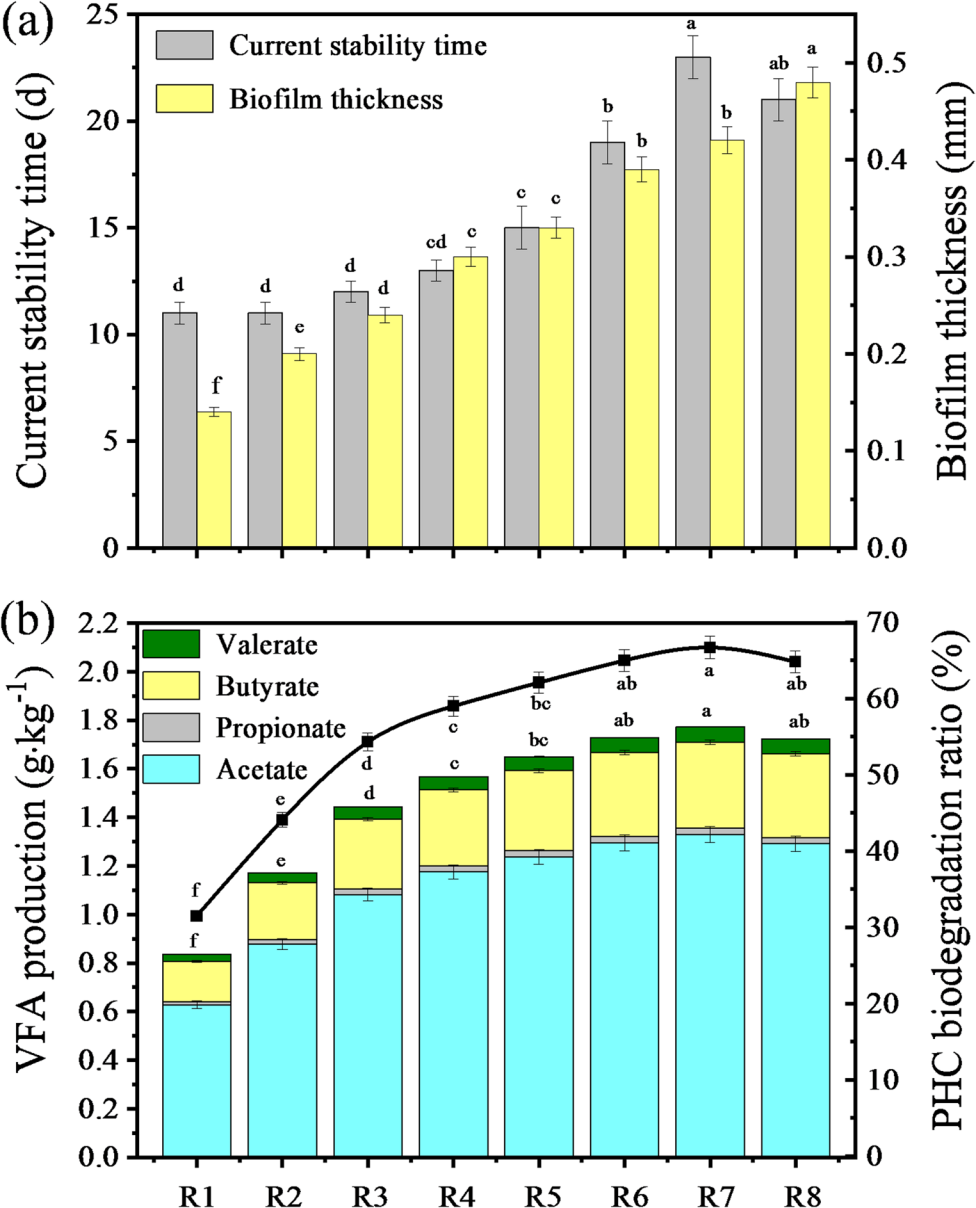
(**a**) Current stabilization time, anodic biofilm thickness, and (**b**) metabolite VFA production and PHC degradation ratio in the reed rhizosphere MFCs with different initial EPS levels. R1-R8 represented the reed rhizosphere MFCs with the initial EPS level from 0 to 128 mg·g^− 1^. Values in the same indicator with different letters indicated significant differences (*P* < 0.05) by Tukey’s honestly significant difference test

All MFC systems operated for 100 days, during which PHC degradation occurred continuously. In the later stages, the degradation slowed significantly due to substrate depletion. As the initial EPS levels increased (0-128 mg·g^− 1^), the total duration of detectable electricity generation in reed rhizosphere MFCs increased from 26 to 51 days, then decreased to 45 days. The stable period of bioelectricity generation extended from 11.0 ± 0.5 days to 23 ± 1 days, then reduced to 21 ± 1 days (Fig. 2a). The stable period of electricity generation performance, which represented the phase of optimal electricity generation, also corresponded to the stable phase of biofilm formation, during which the biofilm thickness reached equilibrium (Fig. 2a). During the stable phase of biofilm formation, the current density, output voltage, coulombic efficiency, power density, current stabilization time, metabolite VFA production and PHC biodegradation ratio displayed a trend of initial increase followed by a subsequent decrease with the elevation of the initial EPS level, reaching their respective peak values at an initial EPS level of 64 mg·g⁻¹. At this optimal EPS level, the corresponding maximum values were 64 ± 1 mA·m^− 2^, 8.04 ± 0.16 V, 60.9 ± 1.2%, 129 ± 3 mW·m^− 2^, 23 ± 1 days, 1.77 ± 0.04 g·kg^− 1^ and 66.7 ± 1.5%, respectively (Figs. 1a and 2a and b). Similarly, the output voltage and power density at a given current density, as reflected in the polarization curve, followed the same trend of first rising and then declining with the initial EPS level (Fig. 1b). The biofilms thickness increased from 0.140 ± 0.005 to 0.480 ± 0.016 mm as the initial EPS level rose (Fig. 2a). Notably, the current stabilization time and biofilm thickness exhibited a more rapid increase compared to other parameters, such as current density, output voltage, coulombic efficiency, power density, metabolite VFA production, and PHC biodegradation ratio, within the range of 0 to 64 mg·g⁻¹ initial EPS levels. Among the VFAs produced as metabolites, acetate and butyrate contents displayed a similar trend of first rising and then falling with the rise in initial EPS level, with their maximum values of 1.33 ± 0.03 g·kg⁻¹ and 0.350 ± 0.009 g·kg⁻¹, respectively, which also observed at 64 mg·g⁻¹. These contents were both significantly higher than those of butyrate and valerate. These findings demonstrated that the long-term stability of biofilm performance in reed rhizosphere MFCs is closely tied to initial EPS level. While moderate EPS level (e.g., 51 days of detectable electricity generation and 23 days of stable bioelectricity production) promoted biofilm development, bioelectricity generation and PHC bioconversion, excessive EPS level led to biofilm overgrowth, increased internal resistance and mass transfer limitations, ultimately reducing the duration and stability of bioelectricity generation (e.g., 45 and 21 days, respectively). This highlighted the importance of optimizing EPS level to sustain biofilm activity and maintain stable performance over extended operation periods in MFC systems.

Previous studies have shown that EPS, as a matrix composed of proteins and polysaccharides, can enhance bacterial adhesion and stabilize biofilm structures, and facilitate electron transfer processes (Ontita et al. 2024; Wang et al. 2024). In this Section, the variations in bioelectricity generation performance and PHC degradation ratio with initial EPS level further highlighted the critical role of EPS in enhancing microbial activity and EET efficiency through redox-active proteins and polysaccharides (Huang et al. 2024; Quan et al. 2024). The increase in biofilm thickness (0.14–0.48 mm) with rising initial EPS level further supported the observation that higher polysaccharide concentrations in EPS contributed to biofilm growth. However, excessive biofilm thickness could lead to increased internal resistance and mass transfer limitations, thereby hindering EET (Qi et al. 2024; Yang et al. 2024; Zhao et al. 2025). This was consistent with the findings of this Section. Additionally, at the optimal EPS level, the PHC biodegradation ratio reached 66.7%, with a power density of 129 mW·m^− 2^, which was comparable to previous studies on PHC degradation in MFCs. Earlier research demonstrated that when PHC underwent biotransformation in MFC systems, the PHC degradation ratio ranged from 15 to 90%, and the maximum power density output varied between 0.08 and 304 mW·m^− 2^ (Lan et al. 2023). The decline in MFC performance at higher EPS level may be attributed to biofilm heterogeneity and bonding degrees, which can negatively impact EET efficiency and microbial activity (Xu et al. 2024). These findings emphasized the importance of optimizing initial EPS level to balance biofilm development and EET efficiency, as excessive EPS can hinder system performance. This study contributed to the growing body of evidence on the role of EPS in bioelectrochemical systems and provided insights into the design and operation of reed rhizosphere MFCs for PHC bioconversion.

### EPS composition and intensity of anodic mature biofilms in reed rhizosphere MFCs with different initial EPS levels

**Fig. 3:**
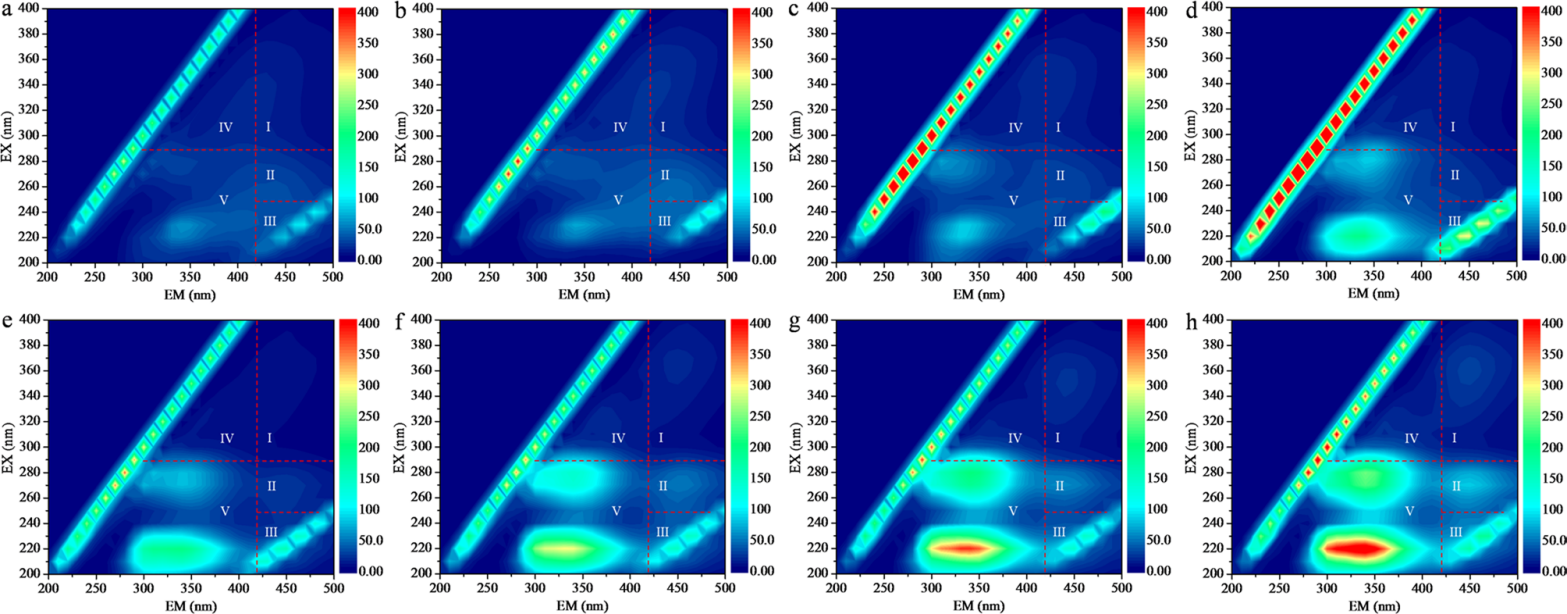
EPS composition and intensity (a.u.) of anodic mature biofilms in the reed rhizosphere MFCs with different initial EPS levels (**a-h**: The biofilm samples in R1-R8)

**Fig. 4:**
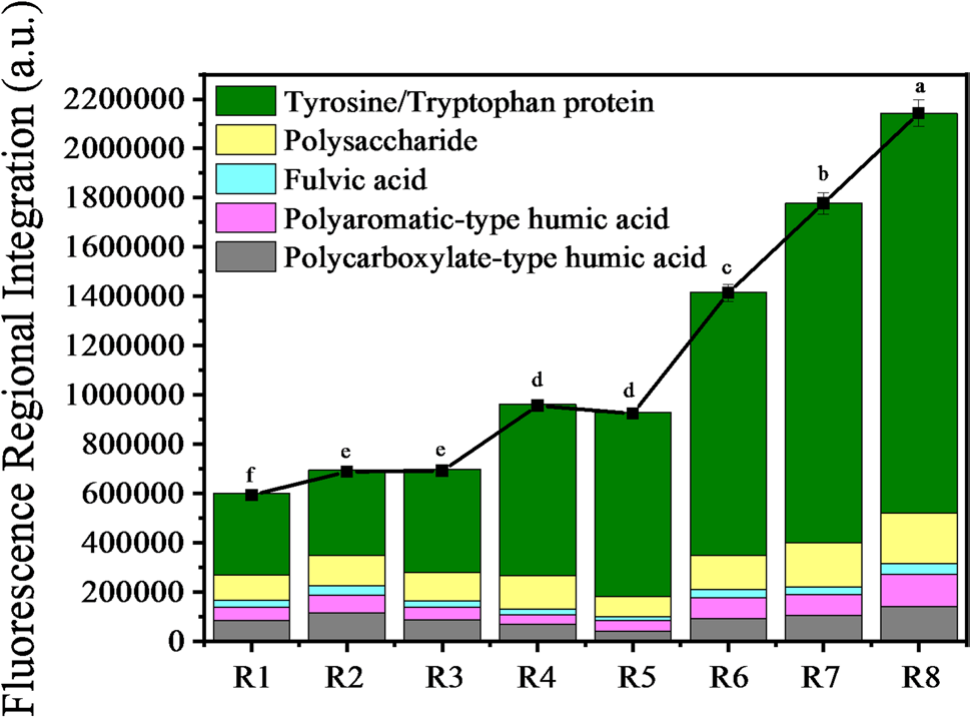
FRI of EPS of anodic mature biofilms in the reed rhizosphere MFCs with different initial EPS levels. R1-R8 represented the reed rhizosphere MFCs with the initial EPS level from 0 to 128 mg·g^− 1^. Values in the same indicator with different letters indicated significant differences (*P* < 0.05) by Tukey’s honestly significant difference test

EPS composition and intensity of anodic mature biofilms during their stable thickness phase in the reed rhizosphere MFCs were displayed in Fig. 3. Proteins having tyrosine-tryptophan, polysaccharide, fulvic acid, polyaromatic-type humic acid and polycarboxylate-type humic acid were identified in regions V, IV, III, II and I, respectively. The fluorescence intensities of proteins having tyrosine-tryptophan, polysaccharide, polycarboxylate-type humic acid, polyaromatic-type humic acid and fulvic acid exhibited a descending trend in the order listed. Unlike the bioelectricity generation and the PHC bioconversion discussed in Sect. Bioelectricity generation and PHC bioconversion in reed rhizosphere MFCs with different initial EPS levels, the fluorescence intensities of polysaccharide and polycarboxylate-type humic acid initially increased, then decreased, and finally increased again with higher initial EPS level. In contrast, proteins having tyrosine-tryptophan, polyaromatic-type humic acid and fulvic acid showed continuous enhancement, correlating with the observed increase in biofilm thickness (from 0.14 to 0.48 mm) (Figs. 2a, 3 and 4). When the initial EPS level increased from 0 to 64 mg·g^− 1^, the trends in proteins having tyrosine-tryptophan, polyaromatic-type humic acid and fulvic acid aligned with changes in biofilm thickness, bioelectricity production, metabolite VFA production and PHC biodegradation (as reported in Sect. Bioelectricity generation and PHC bioconversion in reed rhizosphere MFCs with different initial EPS levels). This suggested that EPS within a certain level drove the formation and the development of anodic biofilms, thereby enhancing the bioelectrical conversion of PHC and bioelectricity production in reed rhizosphere MFCs. However, excessive EPS addition could increase biofilm thickness to 0.48 mm (Fig. 2a), thereby reducing the biofilm permeability and weakening the capacity of material conversion and electron transfer within the anodic biofilms.

The EPS secreted by microorganisms played a pivotal role in protecting cells from unfavorable external environment, forming electroactive biofilms, facilitating substrate biotransformation and driving electron transfer (Gao et al. 2024; Zhao et al. 2021). Extracellular polysaccharides, as a significant component of EPS, contributed to early stages of biofilm formation by adhering to cell surfaces through electrostatic interactions or hydrogen bonding. These polysaccharides formed an interwoven network that provided structural scaffolding for biofilms, enabling cell aggregation, maintaining the integrity of the cell envelope and stabilizing bacterial structures within biofilms (Huang and Puma 2022; Li et al. 2023; Zhu et al. 2022). Studies have shown that mutant biofilms with reduced extracellular polysaccharide production are thinner and exhibit lower cell activity compared to wild-type biofilms. This reduction in extracellular polysaccharides significantly weakened cellular attachment and slowed biofilm development (Zhuang et al. 2022). The underlying mechanism was linked to the decreased content of C-type cytochromes in mutant biofilms, which reduced the electrochemical activity of EPS. Extracellular polysaccharides served as anchoring sites for C-type cytochromes and were essential for efficient EET in electroactive biofilms (Zhuang et al. 2022). While polysaccharides contributed to biofilm thickness and protect cells from external stresses, excessive polysaccharide accumulation could hinder electron transport by increasing the biofilm’s resistance (Yang et al. 2024). This highlighted the importance of balancing EPS composition to optimize biofilm electrochemical performance. For instance, an increased protein-to-polysaccharide (PN/PS) ratio has been shown to stabilize biofilm particle structures and enhance sludge pelletization, further supporting EET processes (Huang et al. 2024).

In mature biofilms, the electrons generated during microbial metabolism must traverse the EPS layer to reach extracellular electrodes. This process was primarily mediated by redox-active substances embedded within EPS (Zhu et al. 2023). Among these substances, extracellular proteins were particularly critical. Proteins such as outer membrane proteins, pili assembly proteins and aggrecan, enabled the storage of electron mediators like cytochromes within the EPS matrix, facilitating EET (Huang and Puma 2022; Li et al. 2023; Zhu et al. 2022). The content of extracellular proteins was highly positively correlated with the EET capacity of biofilms. Redox-active proteins, such as cytochrome C and riboflavin, maintained redox properties between electron acceptors and cells, mediating electron exchange between biofilms and extracellular carriers (Zhao et al. 2025). Furthermore, redox-active proteins in EPS could coordinate with conductive pili to enhance EET efficiency. While conductive pili primarily facilitated electron transfer over longer distances (beyond 10 μm), redox-active proteins dominated electron transfer in regions closer to the electrode (within 10 μm) (Yang et al. 2019). The role of extracellular proteins in biofilm formation and conductivity has been further elucidated through genetic engineering experiments. Overexpression of outer membrane proteins, pili assembly proteins and aggrecan-encoding genes in engineered strains resulted in maximum power densities of 62, 95, and 123 mW/m^2^, respectively, significantly surpassing the original strain’s power density of 30.25 mW/m^2^ (Li et al. 2023). These findings highlighted the ability of extracellular proteins to enhance the redox properties of biofilms and establish an effective electron transport network (Guo et al. 2021). Additionally, EPS contributed to the overall electrochemical performance of biofilms by acting as a reservoir for redox carriers, such as cytochromes, facilitating electron exchange between microorganisms and external electron acceptors (Quan et al. 2024; Wang et al. 2024). The redox-active proteins within EPS not only enhanced the conductivity of the biofilm but also reduced the internal resistance of the microbial cell membrane, thereby improving microbial metabolic activity and EET efficiency (Li et al. 2024). These findings further confirmed that within an appropriate range of initial EPS levels (0–64 mg·g⁻¹), bioelectricity generation performance enhanced as the protein having tyrosine-tryptophan content rose in mature biofilms (Figs. 1, 3 and 4).

In addition, EPS also played a crucial role in solubilizing and increasing the bioavailability of PHCs. Bacterial exo-polysaccharides exhibited amphiphilic properties that allowed them to interface with hydrophobic substrates, effectively mediating the dissolution of poorly-soluble PHCs and enhancing their bioavailability to indigenous microorganisms (Gutierrez et al. 2013). This capability was further supported by the presence of aromatic structures and unsaturated aliphatic chains within EPS, facilitating interactions with molecules containing aromatic rings. Hydrophobic high-molecular-weight polysaccharides distributed on cell surfaces provided binding sites for compounds like phenanthrene and pyrene, enabling their sorption-desorption processes and mass transfer to cells (Wu et al. 2023). These findings further confirmed that within an appropriate range of initial EPS levels (0–64 mg·g⁻¹), PHC biodegradation ratio and metabolite content increased as the EPS content rose in mature biofilms (Figs. 2b, 3 and 4).

### Microbial function of anodic mature biofilms in reed rhizosphere MFCs with different initial EPS levels

The Chao index of anodic biofilm samples initially rose from 3531 to 5149 before dropping to 4849, while the ACE index followed a similar trend, rising from 3652 to 5380 and then declining to 5051 as the initial EPS level increased (0-128 mg·g^− 1^). In contrast, the Simpson index lowered from 0.262 to 0.003, and the Shannon index reduced from 7.07 to 3.71. These results indicated that the richness of microbial communities first increased and then a decreased, whereas its diversity displayed a consistent downward trend with elevated initial EPS levels. This indicated that the addition of EPS enhanced the richness of microbial communities in anodic biofilms within the reed rhizosphere MFCs, while concurrently lessening its diversity.

**Fig. 5:**
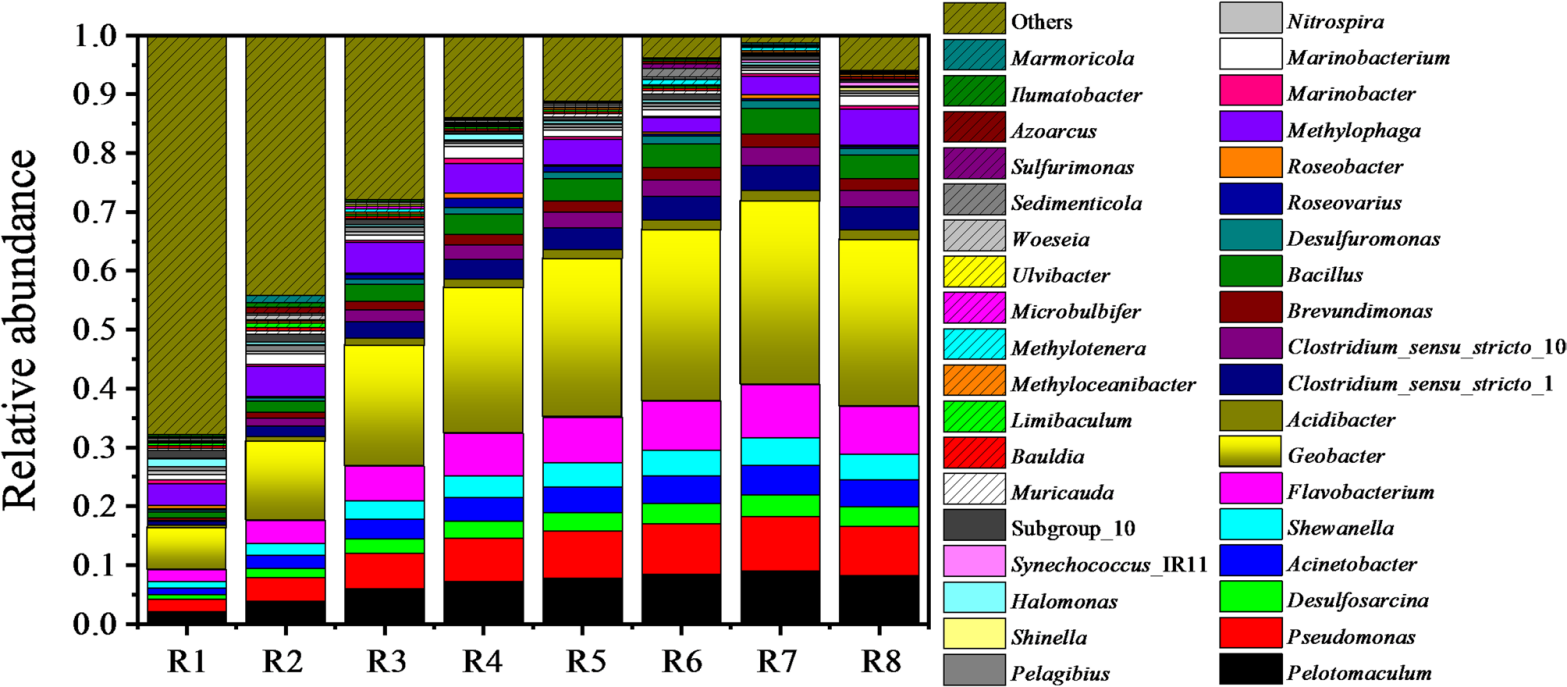
Relative abundance of microbial community of anodic mature biofilms in the reed rhizosphere MFCs with different initial EPS levels. R1-R8 represented the reed rhizosphere MFCs with the initial EPS level from 0 to 128 mg·g^− 1^

The signal exchange mechanism of electroactive biofilms was closely related to their microbial communities (Zhao et al. 2025; Zhu et al. 2023), making it essential to analyze the role of microorganisms in the bioelectrical conversion of PHCs. The relative abundances of microbial communities in anodic mature biofilms from reed rhizosphere MFCs with varying initial EPS levels were exhibited in Fig. 5. The dominant bacterial genera included *Pelotomaculum*, *Pseudomonas*, *Desulfosarcina*, *Acinetobacter*, *Shewanella*, *Flavobacterium*, *Geobacter*, *Acidibacter*, *Clostridium*_*sensu*_*stricto*_1, *Clostridium*_*sensu*_*stricto*_10, *Brevundimonas*, *Bacillus*, *Desulfuromonas*, *Roseovarius*, *Roseobacter*, *Methylophaga*, *Marinobacter*, *Marinobacterium*, *Nitrospira*, *Pelagibius*, *Shinella*, *Halomonas*, etc. Among these, the relative abundances of most bacterial genera presented an initial increase followed by a subsequent decrease as the initial EPS level rose. These trends aligned with the observed changes in bioelectricity production, metabolite production and PHC biodegradation, as discussed in Sect. Bioelectricity generation and PHC bioconversion in reed rhizosphere MFCs with different initial EPS levels.

PHC bioconversion and electricity generation by microorganisms in bioelectrochemical systems (BES) were driven by complex mechanisms involving electroactive and fermentative bacteria. Certain electrochemically active bacteria were capable of directly bio-oxidizing PHCs, playing a critical role in both the initial activation and subsequent oxidation of PHCs. For instance, the electroactive microorganism *Geobacter metallireducens* was reported to catalyze the complete electrooxidation of monoaromatic hydrocarbons such as toluene, phenol and benzene (Gebregiorgis Ambaye et al. 2023). Another important group involved in PHC bioconversion in BES were sulfate-reducing bacteria (SRB). In these systems, the anode facilitated the biotic or abiotic trans-oxidation of sulfide to sulfate, regenerating the metabolic electron acceptor. SRB, such as *Desulfosarcina* and *Desulfuromonas*, reduced the regenerated sulfate, which served as an electron acceptor to aid in PAH biodegradation (Gebregiorgis Ambaye et al. 2023). More importantly, the synergistic interaction between fermentative and electroactive bacteria played a pivotal role in bioelectric PHC conversion (Zhao et al. 2025). Fermentative bacteria partially oxidized PHCs to VFAs, CO_2_, H_2_ and their mixtures through reactions such as fumarate addition, carboxylation or hydroxylation. These intermediates were subsequently oxidized by *Geobacter* species, which utilized the electrode as a terminal electron acceptor, facilitating rapid electron transfer (Zhao et al. 2023, 2025). This cooperation not only enhanced PHC biodegradation but also ensured thermodynamic stability throughout the reaction sequence (Tucci et al. 2021). Key bacterial genera identified in this study, including *Pelotomaculum*, *Pseudomonas*, *Desulfosarcina*, *Acinetobacter*, *Shewanella*, *Flavobacterium* and *Geobacter*, exhibited both bioelectrochemical activity and PHC degradation capability (Meng et al. 2024; Zhao et al. 2023). Their bioelectrochemical activity was mediated through mechanisms such as direct or indirect electron transfer via conductive pili, self-produced C-type cytochromes or exogenous electron-shuttling molecules (Zhao et al. 2025). EPS further contributed to the process by promoting microbial adhesion, biofilm formation, and facilitating electron transfer through intermediary moleculars, self-conducting structures or direct interspecies (Zhao et al. 2023). Additionally, the consistency between the changes in the relative abundances of these functional bacteria and the variations in bioelectricity generation, metabolite production and PHC biodegradation further revealed that EPS enriched and activated these functional bacteria, thereby promoting bioelectricity generation and PHC bioconversion. This underscored the critical role of EPS in shaping microbial community structure and enhancing electron transfer efficiency through biofilm formation and stabilization.

**Fig. 6:**
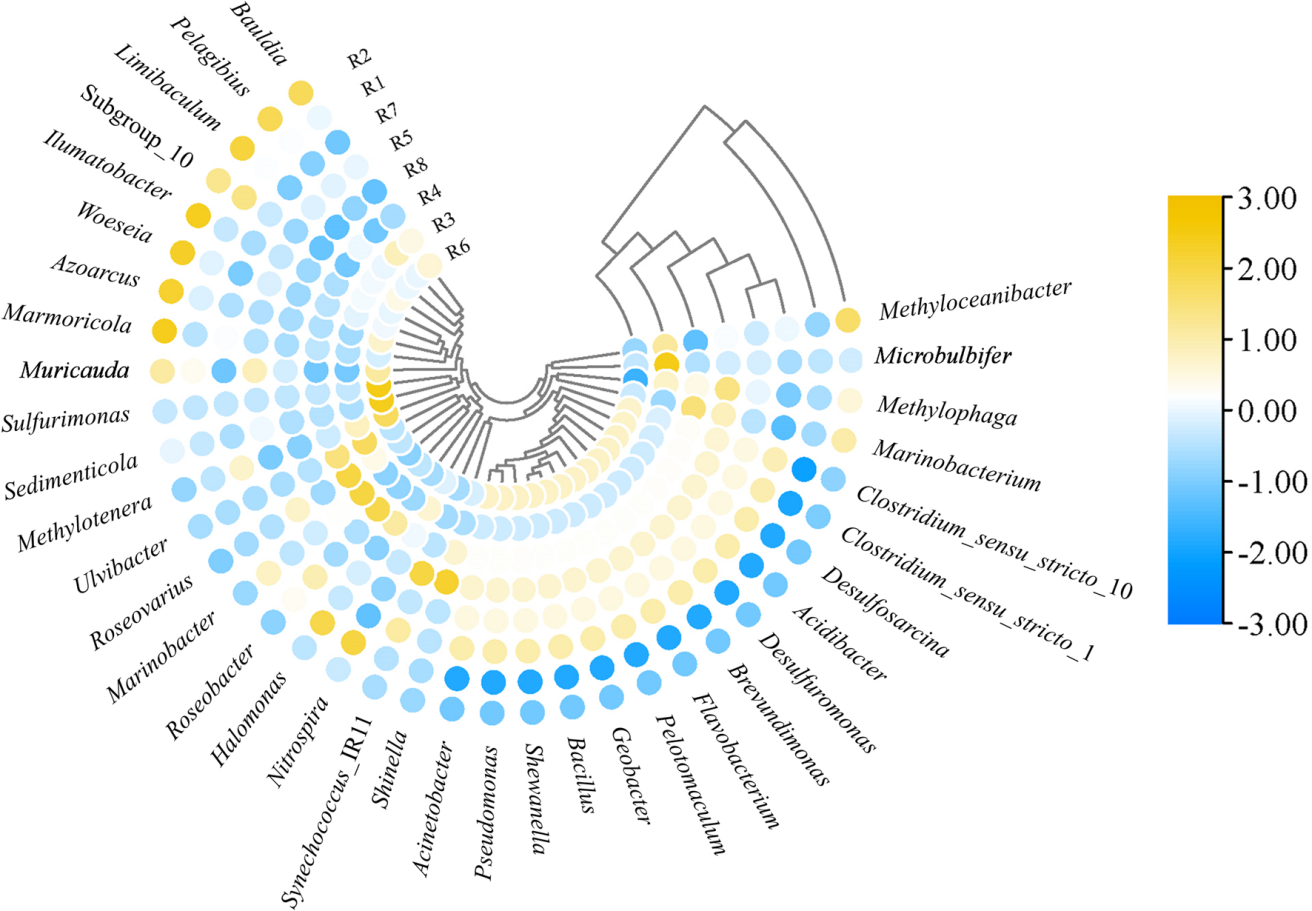
Heatmap of microbial community of anodic mature biofilms in the reed rhizosphere MFCs with different initial EPS levels. R1-R8 represented the reed rhizosphere MFCs with the initial EPS level from 0 to 128 mg·g^− 1^

The heatmap of microbial community of anodic mature biofilms in reed rhizosphere MFCs with different initial EPS levels was presented in Fig. 6. Samples R1 and R2 demonstrated a close genetic relationship, while samples R3-R8 exhibited relatively similar affinities. The functional bacteria with close affinities included *Pelotomaculum*, *Pseudomonas*, *Desulfosarcina*, *Acinetobacter*, *Shewanella*, *Flavobacterium*, *Geobacter*, *Acidibacter*, *Clostridium*_*sensu*_*stricto*_1, *Clostridium*_*sensu*_*stricto*_10, *Brevundimonas*, *Bacillus* and *Desulfuromonas*. These bacteria showed a trend of initially increasing and then decreasing abundance with elevated initial EPS level (Fig. 5), consistent with the findings in Sect. Bioelectricity generation and PHC bioconversion in reed rhizosphere MFCs with different initial EPS levels. The addition of EPS significantly influenced microbial community dynamics, promoting the enrichment of closely related functional bacteria, thereby enhancing community richness while reducing diversity. This phenomenon was supported by the findings from studies conducted in PHC-polluted seawater, where the addition of EPS induced notable shifts in microbial community richness and diversity, significantly promoting the proliferation of genera such as Pseudomonas and Bacillus (Meng et al. 2024). This stimulation was accompanied by a marked rise in cytochrome P450 gene copies, as well as the upregulation of genes associated with ethanol dehydrogenation. Furthermore, EPS addition promoted the activity of key enzymes, including lipase, phosphatase, ethanol dehydrogenase and alkane hydroxylase (Meng et al. 2024). These findings suggested that EPS not only served as a potential substrate or nutrient source for microbial growth and activity but also acted as a stimulant, boosting microbial metabolic activity and functional capacity. This dual role of EPS likely explains its ability to enrich and activate specific functional bacterial groups in microbial communities, and this enrichment and activation of electrochemically active and PHC-degrading bacteria are key to achieving efficient PHC bioconversion and bioenergy recovery in reed rhizosphere MFCs.

### The network correlation of functional microorganisms with bioelectricity generation, PHC bioconversion and EPS intensity in reed rhizosphere MFCs with different initial EPS levels

**Fig. 7:**
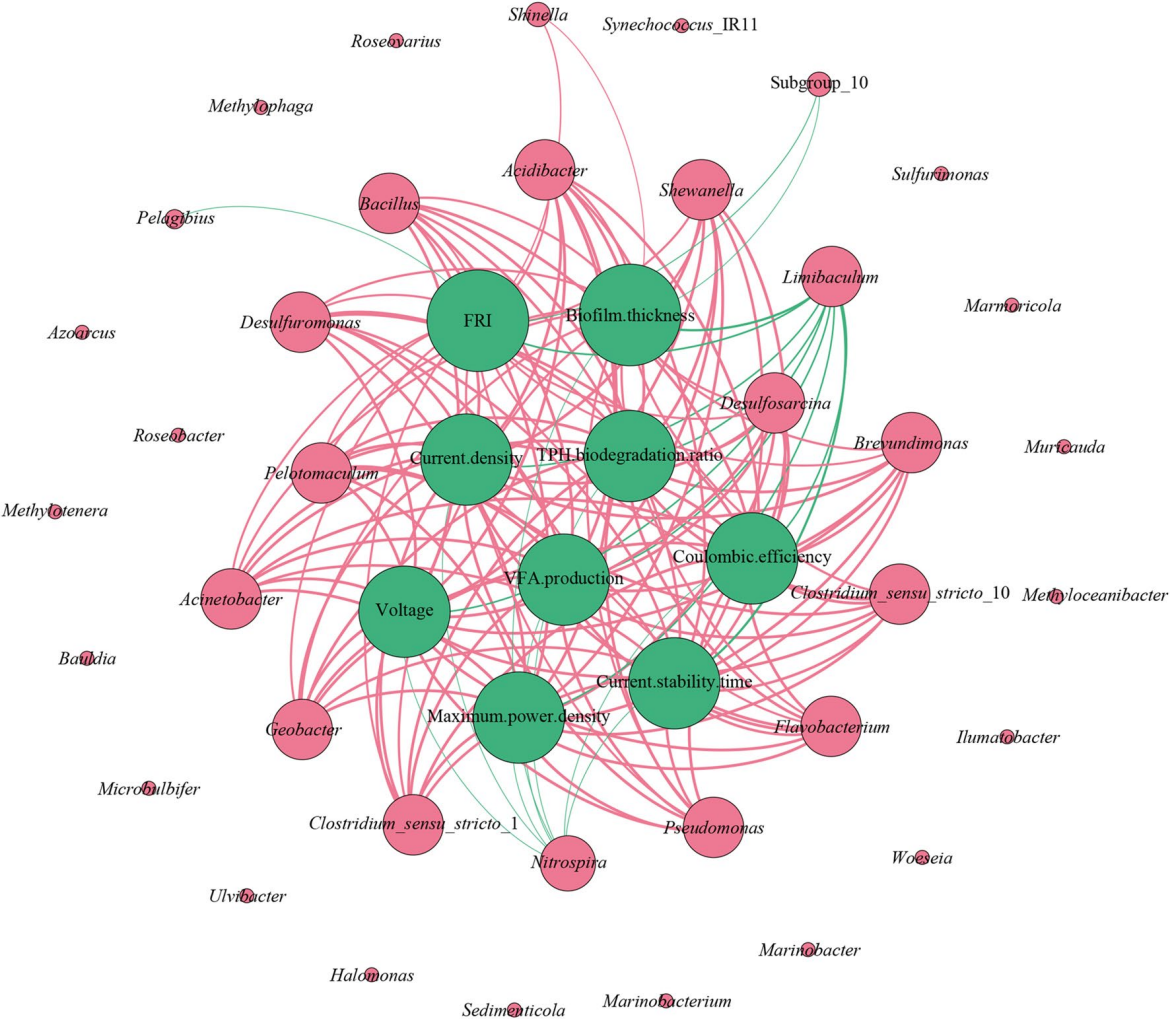
Network association of functional microorganisms with PHC biodegradation ratio, metabolite VFA production, current density, output voltage, coulombic efficiency, maximum power density, current stability time, biofilm thickness and FRI of EPS in the reed rhizosphere MFCs with different initial EPS levels

The functional bacteria capable of dual functions-PHC bioconversion and bioenergy recovery-included *Pelotomaculum*,* Pseudomonas*, *Desulfosarcina*, *Acinetobacter*, *Shewanella*, *Flavobacterium*, *Geobacter*, *Acidibacter*, *Clostridium*_*sensu*_*stricto*_1, *Clostridium*_*sensu*_*stricto*_10, *Brevundimonas*, *Bacillus* and *Desulfuromonas* (as reported in Sect. Microbial function of anodic mature biofilms in reed rhizosphere MFCs with different initial EPS levels). Under varying initial EPS levels, the relative abundances of these functional bacteria showed strong positive correlations with PHC biodegradation ratio, metabolite VFA production, current density, output voltage, coulombic efficiency, maximum power density, current stability time, biofilm thickness and FRI of EPS. These correlations further confirmed that these bacteria were closely linked to bioelectrical PHC conversion and bioelectricity production in reed rhizosphere MFCs (Fig. 7). Based on the results obtained in this study and the previous studies, EPS within certain levels drove the enrichment of electrochemically active and PHC-degrading bacteria with close affinities, promoting microbial community richness while reducing its diversity in reed rhizosphere MFCs (as reported in Sect. Microbial function of anodic mature biofilms in reed rhizosphere MFCs with different initial EPS levels). These functional bacteria induced the formation and development of anodic biofilms, particularly increasing the content of proteins having tyrosine-tryptophan, which were crucial in the electron transport network of anodic biofilms (as reported in Sect. EPS composition and intensity of anodic mature biofilms in reed rhizosphere MFCs with different initial EPS levels). The stable anodic biofilms facilitated bioelectrical PHC conversion through the metabolism of PHC-degrading bacteria and enhanced bioelectricity production via electron transfer by electrochemically active bacteria, utilizing their own conductive pili, self-produced C-type cytochromes or exogenous electron shuttling molecules (as reported in Sect. Bioelectricity generation and PHC bioconversion in reed rhizosphere MFCs with different initial EPS levels). Additionally, some bacteria acted antagonistically to these functional bacteria. For example, the relative abundances of *Nitrospira*, *Pelagibius*, Subgroup_10 and *Limibaculum* were strongly negatively correlated with bioelectricity production, metabolite VFA production and PHC biodegradation (Fig. 7), suggesting an antagonistic role for these bacteria against the fermentative and electrochemically active bacteria. Different initial EPS levels drove these bacteria to perform various functions, collectively contributing to the stabilization of microbial community in reed rhizosphere MFCs. These findings illustrated that the dual role of EPS was crucial for the enrichment and activation of specific functional bacterial groups within microbial communities in reed rhizosphere MFCs. The positive correlations between the relative abundances of these bacteria and various metrics of bioelectricity production and PHC bioconversion highlighted their integral role in these processes. Furthermore, Different initial EPS levels influenced the balance between functional and antagonistic bacteria, ultimately contributing to the stability and efficiency of the microbial ecosystem.

### Challenges and future research directions

Despite the superior attractiveness of EPS-driven MFCs for remediation of PHC-contaminated soils, challenges related to scalability, efficiency and long-term operation remain. The low power density of MFCs limits scalability, while the complex composition of PHC-contaminated soils may disrupt biofilm stability and electron transfer efficiency. Scaling up MFCs introduces issues such as uneven pollutant distribution, altered internal resistance, and reduced microbial-electrode interactions (Lan et al. 2023). Additionally, bioelectrochemical remediation rates are slower than chemical methods, and long-term operation efficiency remains a concern (Mahto and Das 2025). Addressing these challenges requires interdisciplinary integration in electrode material development, microbial enrichment and system design to improve scalability, efficiency and cost-effectiveness, thereby achieving sustainable environmental and energy goals.

Future research should prioritize the development of advanced materials and bioengineering approaches to improve the performance of EPS-driven MFCs. For example, biocompatible electrode materials with high conductivity and surface area can promote biofilm growth and electron transfer (Mahto and Das 2025). Additionally, selective enrichment of electroactive and hydrocarbon-degrading microbial communities is necessary to adapt MFCs to diverse environmental conditions. Genetic engineering and synthetic biology can also play a role in enhancing the metabolic capabilities of key microbial species, thereby improving both power output and substrate utilization (Mahto and Das 2025; Zhao et al. 2025). Interdisciplinary collaboration will be crucial for addressing these challenges and advancing the field of bioelectrochemical remediation. Insights from electrochemistry, soil science, microbiology, and material engineering can inform the design of more efficient and cost-effective systems (Lan et al. 2023). Moreover, integrating EPS-driven MFCs with other renewable energy technologies, such as wind or solar power, can pave the way for hybrid systems capable of generating sustainable energy while simultaneously addressing environmental pollution (Mahto and Das 2025). In addition to their role in remediation, EPS-driven MFCs also hold potential as biosensors for real-time monitoring of hydrocarbons and other contaminants, further expanding their utility in environmental applications (Lovecchio et al. 2024).

## Conclusions

This research underscored the crucial role of EPS in driving the bioelectrical conversion of PHCs through rhizosphere MFCs, enabling the clean conversion of PHCs into bioenergy. Key performance indicators including current density, output voltage, coulombic efficiency, maximum power density, current stabilization time, metabolite VFA production and PHC biodegradation ratio, exhibited an initial increase followed by a decline, peaking at an optimal EPS level of 64 mg·g⁻¹. Fluorescence intensity of proteins having tyrosine-tryptophan showed a continuous increase, aligning with the observed biofilm thickness trend (from 0.140 ± 0.005 to 0.480 ± 0.016 mm). The relative abundances of key bacterial genera, including *Pelotomaculum*, *Pseudomonas*, *Desulfosarcina*, *Acinetobacter*, *Shewanella*, *Flavobacterium*, *Geobacter*, *Acidibacter*, *Clostridium*_*sensu*_*stricto*_1, *Clostridium*_*sensu*_*stricto*_10, *Brevundimonas*, *Bacillus* and *Desulfuromonas*, followed a similar trend, increasing initially and then decreasing with rising EPS level. These dominant bacteria acted as both electrochemically active bacteria and PHC-degrading bacteria, performing a dual role in bioelectricity generation and PHC conversion. This research provides valuable insights and practical guidance for optimizing PHC-to-bioenergy conversion in rhizosphere MFCs and refining the role of EPS in bioelectrical PHC conversion, particularly in petroleum-contaminated wetlands of the Yellow River Delta.

## Data Availability

The datasets and materials used and/or analyzed during the current study are available from the corresponding author on reasonable request.
